# FNAC of Bacillus- Calmette- Guerin lymphadenitis masquerading as Langerhans cell histiocytosis.

**DOI:** 10.1186/1742-6413-1-6

**Published:** 2004-12-21

**Authors:** Nalini Gupta, Vijay Kumar, Raje Nijhawan, Radhika Srinivasan, Arvind Rajwanshi

**Affiliations:** 1 Department of Cytopathology and Gynecological Pathology, Postgraduate Institute of Medical Education and Research, Chandigarh, India

## Abstract

Bacillus Calmette Guerin (BCG) lymphadenitis is a well known entity. Disseminated BCG infection usually presents as generalized lymphadenopathy, skin rash and hepatosplenomegaly and at times, can pose a diagnostic challenge to clinicians. There are only a few published studies on the cytological findings of BCG lymphadenitis. In this letter we report the fine needle aspiration cytology (FNAC) of BCG lymphadenitis clinically masquerading as Langerhans cell histiocytosis (LCH). FNA smears showed sheets of foamy macrophages and many polymorphs in a dirty necrotic background with many macrophages as well as polymorphs showing negatively stained rod like structures within their cytoplasm. Zeihl Neelson stain revealed that these cells were heavily loaded with acid fast bacilli (AFB). In the index case, AFB were also seen within the cytoplasm of polymorphs, which has not been documented earlier in the literature.

## To the Editor

**Figure 1:**
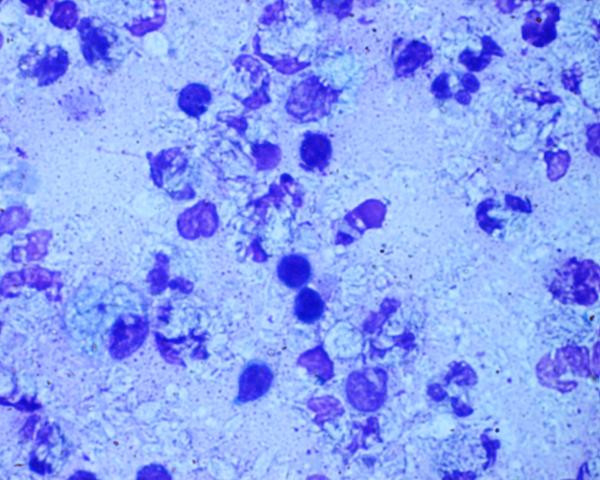
Microphotograph showing many macrophages with negative shadows and polymorphs in a necrotic background (MGG X 1375).

**Figure 2:**
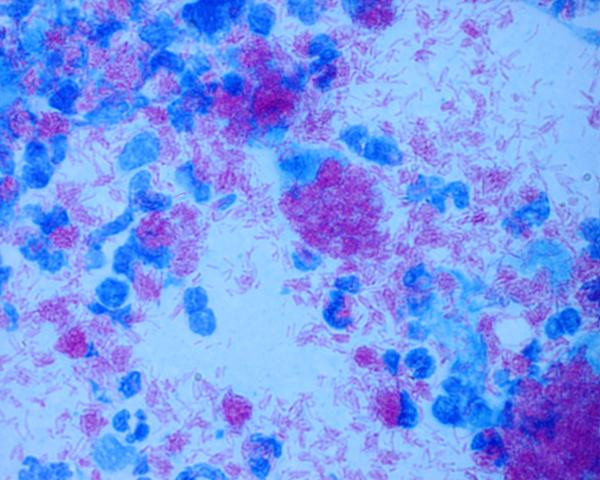
Microphotograph showing extensive load of acid fast bacilli both intracellularly (in macrophages as well as polymorphs) and extracellularly (Z-N Stain X 1375).

Bacillus Calmette Guerin (BCG) vaccine has been included in the immunization programmes of the developing countries including India. Immunization with BCG is done on the first day of life. The most common response to BCG vaccine is sub clinical. However, some infants may develop regional lymphadenitis or a subcutaneous abscess at the vaccination site [1]. Disseminated BCG infection usually presents as generalized lymphadenopathy, skin rash and hepatosplenomegaly [2]. There are only a few published studies on the cytological findings of BCG lymphadenitis [3-5]. In this letter we report the Fine needle aspiration cytology (FNAC) of BCG lymphadenitis clinically masquerading as Langerhans cell histiocytosis (LCH). This index case is from a two and a half month old male child, who presented with generalized lymphadenitis, skin rash and delay in developmental milestones. On examination, moderate hepatosplenomegaly was noted. The patient had been immunized properly for his age. The clinical possibility of LCH was strongly considered. On hematological examination, the counts were within normal limits. Fine needle aspiration was performed on a 3 cm, soft, left axillary lymph node. Thick pus was aspirated and the swelling reduced in size after aspiration. A part of the aspirated material was sent for mycobacterial (AFB) culture. May- Grunwald Geimsa (MGG), Hematoxylin and Eosin and Ziehl Neelson (ZN) stains were performed on the smears. Microscopically, sheets of foamy macrophages lying singly and in clusters were noted along with many polymorphs and nuclear debris in a dirty necrotic background. No epithelioid cell granulomas were seen. Many of the macrophages showed negatively stained (unstained) rod like structures within their cytoplasm (pseudo Gaucher cells). Similar structures were identified extracellularly, as well as within the polymorphs (Figure 1). Streaked cytoplasm was also noted. On Z-N staining, sheets of AFB positive bacilli were seen both intracellularly (in macrophages as well as polymorphs) as well as extracellularly. They corresponded with the negative shadows seen with MGG staining (Figure 2). The culture for mycobacteria was negative. The diagnosis of BCG lymphadenitis was offered and it was advised to further investigate the patient for immunodeficiency states. Later, a 1 cm right axillary lymph node was also aspirated. It revealed reactive hyperplasia microscopically but showed strong AFB positivity.

Enlargement of regional lymph nodes is a well-known complication of BCG vaccination and occurs within 6 months of vaccination [3]. It may be suppurative or non-suppurative and smears show a combination of necrosis and epithelioid cell granulomas [3-5]. Acid-fast bacilli are demonstrable in nearly all the cases. Disseminated BCG infection can however, pose a diagnostic challenge to clinicians (as happened in the index case). The occurrence of systemic manifestations like skin rash and hepatosplenomegaly can closely mimics those of hematological malignancies. Smears from these cases show predominantly histiocytes in a background of polymorphs, and debris heavily loaded with AFB. In the index case, AFB were also seen within the cytoplasm of polymorphs, which has not been documented earlier in the literature. However, this observation could also be due to overlap of polymorphs by numerous extracellular organisms present in the background. This can definitely be commented upon only by electron microscopic examination, which was not carried out in this case. This might also be related to the type and extent of immunological deficiency in the patient. Moreover, the case was also deceptive from the clinical view point, as it closely mimicked LCH. As such, this case further reinforces the role of FNAC as a diagnostic modality.
